# High-Volume Phosphogypsum Cement Stabilized Road Base: Preparation Methods and Strength Formation Mechanism

**DOI:** 10.3390/ma17246201

**Published:** 2024-12-19

**Authors:** Meng Zou, Zhaoyi He, Yuhua Xia, Qinghai Li, Qiwen Yao, Dongwei Cao

**Affiliations:** 1 School of Civil Engineering, Chongqing Jiaotong University, Chongqing 400074, China; 622220951078@mails.cqjtu.edu.cn (M.Z.); 622230951035@mails.cqjtu.edu.cn (Y.X.); qinghaili2002@163.com (Q.L.); 2 School of Traffic and Transportation, Chongqing Jiaotong University, Chongqing 400074, China; 3 China Metallurgical Construction Engineering Group Company Limited, Chongqing 400084, China; yqwgzyx@sina.com; 4 Research Institute of Highway Ministry of Transport, Beijing 100088, China; caodongwei@vip.126.com

**Keywords:** high-volume phosphogygpsum, cement stabilized road base, strength formation mechanism, leaching toxicty

## Abstract

This study investigated the potential for efficient and resourceful utilization of phosphogypsum (PG) through the preparation of a High-volume Phosphogypsum Cement Stabilized Road Base (HPG-CSSB). The investigation analyzed the unconfined compressive strength (UCS), water stability, strength formation mechanism, microstructure, and pollutant curing mechanism of HPG-CSSB by laser diffraction methods (LD), X-ray diffraction (XRD), fourier-transform infrared spectroscopy (FTIR), scanning electron microscopy (SEM), energy-dispersive X-ray spectroscopy (EDS), and inductively coupled plasma-mass spectrometry (ICP-MS). The optimal mix ratio of HPG-CSSB was 4% cement, 1% CA2, 35% PG, and 60% graded crushed stone. The UCS reached 6.6 MPa, 9.3 MPa, and 11.3 MPa at 7, 28, and 60 d, respectively. The alkaline curing agent stimulated cement activity and accelerated the release of Ca^2+^ and SO_4_^2−^ from the PG. This formed many C-S-H gels and ettringite (AFt). The curing agent converted Ca^2+^ to C-(A)-S-H gels due to high volcanic ash activity. The diverse hydration products strengthened HPG-CSSB. The HPG-CSSB exhibits favorable water stability, demonstrating a mere 7.6% reduction in strength following 28 d of immersion. The C-S-H gel and AFt generated in the system can carry out ion exchange and adsorption precipitation with F^−^ and PO_4_^3−^ in PG, achieving the curing effect of toxic and hazardous substances. HPG-CSSB meets the Class A standard for integrated wastewater discharge.

## 1. Introduction

Phosphogypsum (PG) is a by-product of phosphate fertilizer and phosphoric acid industrial production.The main component is the inorganic compound calcium sulfate (CaSO_4_), which constitutes approximately 80% of the total amount of PG [1,2]. Further, PG comprises a range of impurities, including silicon oxides (SiO_2_), alumina (Al_2_O_3_), iron oxides (Fe_2_O_3_), soluble phosphates (PO_4_^3−^), fluoride (F^−^) and several heavy metal elements (Mg^2+^, As^3+^, Pb^2+^) [3], in addition to naturally occurring radioactive elements such as ^40^K, ^238^U and ^232^Th [4]. With global reserves estimated at approximately 6 billion tons, PG is widely distributed throughout the world. In 2023, approximately about 300 million tons of new PG were produced worldwide [5,6], of which 81 million tons were produced in China. Nevertheless, the overall utilization rate of PG in a range of applications is only 15% [7,8], with the annual utilization of PG significantly lower than the global production. Currently, the majority of PG is still stored in open piles for extended periods, which not only results in the wastage of considerable land resources but also leads to the contamination of soil and groundwater with toxic and harmful substances through rainwater leaching, causing significant environmental harm.

Presently, the comprehensive utilization of PG is dominated by its use as a cement retarder, in road-building materials, for ecological restoration, and in construction gypsum [9]. Additionally, it is employed as a mine-filling material, in gypsum boards, as a raw material for sulfuric acid production, in gypsum bricks, in road-building materials, and in gypsum blocks, among other applications. Among these applications, the use of PG as a road-building material allows for efficient consumption [10]. Shen et al. [11] prepared an all-solid waste road base material with an optimum mix ratio of fly ash to steel slag (50:50) and an additional 2.5% PG. The 7 d unconfined compressive strength (UCS) of the aforementioned mix ratio was 1.86 MPa, which is considerably below the standard for cement-stabilized crushed stone road bases. However, it is in compliance with the standard for lime fly ash road bases as outlined in the relevant specification. After 28 days of curing, the UCS increased to 8.36 MPa, and the long-term durability was also enhanced, with a strength loss of only 2.35% after 25 wet and dry cycles. Ghosh et al. [12] investigated the compaction and bearing ratio characteristics of pond ash stabilized with lime alone or in combination with PG. The findings indicated that the incorporation of a modest quantity of PG (0.5% or 1.0%) enhanced the bearing ratio of the pond ash-stabilized material and acted as a curing agent. Dutta et al. [13] prepared fly ash stabilized materials with 8% lime and 2% PG and cured them for 7 d–90 d using different curing methods. The results demonstrated a notable increase in deviator stress, cohesion, friction angle, initial tangent modulus, and secant modulus at 28 d. This stabilization system facilitates the utilization of PG in a range of geotechnical applications. In a recent study, Kate D. Weiksnar et al. [14] investigated the radionuclide problem present in PG used in road construction. The researchers examined the effectiveness of incorporating PG with limestone rock asphalt (LR) or recycled concrete aggregate (RCA) in the preparation of road base materials in reducing the mobility of radionuclides. The study demonstrated that mixing was an effective method for reducing the mobility of radionuclides, including radium-226 (Ra-226). However, the effectiveness of mixing was found to be influenced by the age of the PG and the type of material utilized. Nevertheless, the overall outcome was that the mobility of radionuclides was maintained below the regulatory threshold. This study confirms that the environmental problems caused by the use of high dosages of PG for road construction can be solved.

If PG is employed solely as an admixture, it will prove challenging to resolve the prevailing issue of low total utilization of PG on a global scale. Consequently, an increasing number of researchers are exploring the use of higher doses of PG in road materials. For instance, Gregory et al. [15] prepared stabilized PG materials with fly ash or cement using two types of PG with different pH values. The findings indicated that the compressive strength of the stabilized materials was contingent upon the pH of the PG. Specifically, the compressive strength of stabilized materials utilizing PG (pH ≈ 5) combined with 30% fly ash or 10% cement could satisfy the requisite specifications. However, a lower pH PG (pH ≈ 3) would markedly diminish the strength of the material, rendering it unsuitable for its intended use. Moreover, some researchers have proposed that PG can be modified with lime to enhance its properties, thereby facilitating its utilization in road construction. Subsequently, scholars began to investigate the use of PG in conjunction with high volcanic ash activity materials. A substantial body of research was conducted on phosphogypsum lime fly ash (PLF) mixtures, which yielded certain engineering outcomes [16,17,18]. Amrani et al. [10] prepared a mixture of PG with clay soil, fly ash, lime, and calcareous material for use as a paving material for road bases and sub-bases. The optimum mix ratio was found to be (calcareous material:PG:fly ash):hydraulic road binder = (10:25:65):7. It was observed that the mechanical properties at the optimum mix ratio could be enhanced by means of improving the alkalinity of the mixture. This is due to the fact that higher alkalinity is conducive to the formation of ettringite (AFt), which has been demonstrated to improve the mechanical properties of the mixture.

Additionally, PG has the capacity to react with cement, forming AFt and thereby enhancing the pre-existing strength [19]. Consequently, the utilization of PG in cement-stabilized materials is a subject of considerable research interest. Wan et al. [20] used Bayer red mud and PG in cement-stabilized dredged soil. Their findings indicated that the alkaline additive activated the volcanic ash minerals in the dredged soil, resulting in the production of substantial quantities of AFt at the optimum mix ratio of Bayer red mud to PG (50:50). Wu et al. [21] subjected PG to a pretreatment involving washing and calcination and the use of sodium metasilicate nonahydrate (SMN) as a curing agent to enhance the strength of materials. The results demonstrated that the pretreatment could moderately diminish the impact of PG on the base material’s properties. However, the laborious nature of the pretreatment and the high dosage of the curing agent limited the feasibility of its extensive practical application. Sun et al. [22] prepared high-volume PG and graded crushed stone stabilized materials using cement and fly ash as inorganic binders. The optimum mix ratio of cement, fly ash, PG, and gravel was proposed as 6:5:50:39, with 0.3% of activator mixed in. The 7 d UCS was 4.1 MPa, which is in accordance with the requisite strength standard for a highway base layer. In this study, a pretreatment process involving the mixing of quicklime and washing was employed for PG, thereby further enhancing the dosage of PG in the stabilized material. Nevertheless, the strength gap compared to conventional cement-stabilized crushed stone base remains considerable. Liang et al. [23] developed a stabilized base comprising excess-sulfate phosphogypsum slag cement (ESPSC) and phosphogypsum-based artificial aggregate, which could significantly enhance the utilization of PG, exceeding 40%. However, this approach did not yield enhanced strength performance, with the 7 d UCS with the optimum ratio reaching only 3.02 MPa.

While the PG-stabilized materials have yielded a substantial number of results across a range of research, there are still some limitations to be addressed, particularly in terms of economic feasibility and the complexities associated with practical construction techniques, which seem to have been given less emphasis than they deserve, but this is closely related to the resourceful use of PG. Accordingly, this study draws on the design methodology of an inorganic binder comprising cement, raw PG, and high volcanic ash-activated materials to prepare a High-volume Phosphogypsum Cement Stabilized Road Base (HPG-CSSB). This approach aims to reduce the quantity of crushed stones in the stabilized material through the use of 25–45% raw PG, limit the amount of cement to the general amount utilized in subgrade construction to enhance economic efficiency and employ only a modest amount of slag-based curing agents CA1 and CA2 to augment the strength characteristics of stabilized materials. The impact of varying inorganic binder percentages, differing cement PG ratios, and diverse curing agent types and concentrations on the strength characteristics and water stability of HPG-CSSB were examined. Moreover, the hydration mechanism and microstructure of the interfacial transition zone (ITZ) of HPG-CSSB were investigated through X-ray diffraction (XRD), Fourier transform infrared spectroscopy (FTIR), and scanning electron microscopy with energy-dispersive X-ray spectroscopy (SEM-EDS) analysis. Furthermore, the leaching toxicity and heavy metal curing mechanism were examined using ICP-MS. In conclusion, the objective of this study is twofold: firstly, to prepare a new type of PG-stabilized pavement base materials; secondly, to elucidate the strength formation mechanism of the materials at multiple scales, with the aim of effectively improving the utilization of PG.

## 2. Materials and Methods

### 2.1. Raw Materials

**Figure 1:**
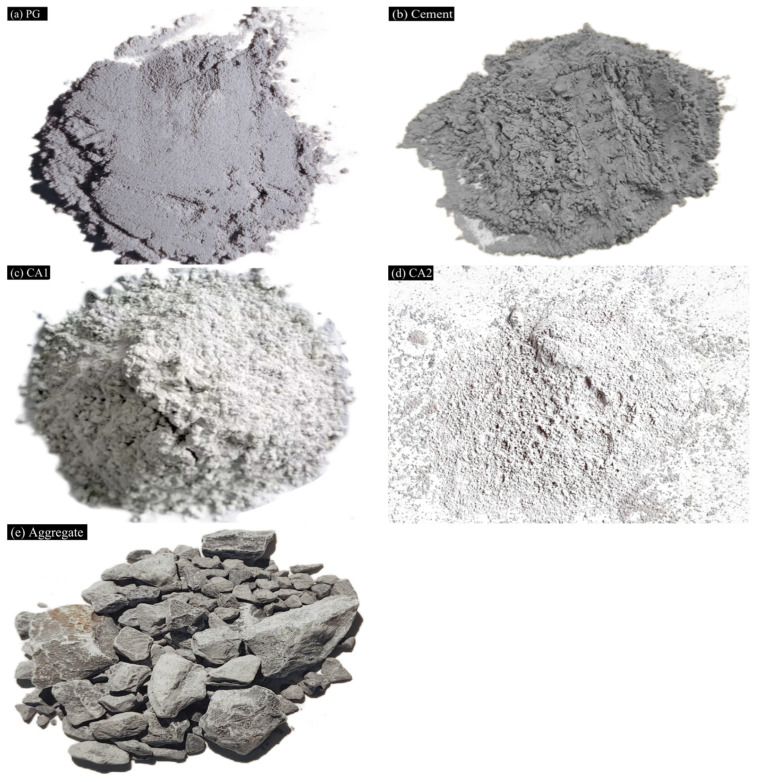
Raw material morphology.

The phosphogypsum (PG) collected from a yard in Sichuan Province, China, was observed to have a grayish-white coloration and a pH of 5.4. The cement was a 42.5-grade ordinary silicate cement procured from a cement factory in Chongqing, China, and it was a gray substance. The primary components of the curing agents are slag, silica fume, and sodium hydroxide, among others. The aggregate is limestone, and its related technical indexes meet the requirements set forth in JTG/E42-2005, “Test Methods of Aggregate for Highway Engineering”. [24] The grading design is presented in Table 1. The morphology of all raw materials is illustrated in Figure 1.

### 2.2. Mix Proportion Design

In this test, the proportion of inorganic binders was set at 30%, 40%, and 50%, with the cement PG ratio established at 1:5, 1:6, 1:7, 1:8, and 1:9. The proportion of graded crushed stone was set at 70%, 60%, and 50%, and the amount of water added to each ratio was determined by the compaction test. Subsequently, the 7 d UCS was employed as the criterion for selecting the preferable mixture.

To enhance the strength characteristics of the samples, two supplementary curing agents were employed to cure the materials in this investigation. To investigate the synergistic relationship between the curing agents and the cement and to ensure the economy and practicability of HPG-CSSB, the cement in the optimum mixture was replaced with curing agents CA1 and CA2 in additional ratios, with the substitution rates set to 0%, 20%, 40%, 60%, 80%, and 100%, respectively. This was carried out in order to determine the optimal ratio of curing agent to cement for the mixture. The specific designs are illustrated in Table 2 and Table 3.

### 2.3. Proctor Compaction Test

In accordance with T 0804-1994, “Compaction Test Method of Inorganic Binder Stabilizing Material”, as outlined in JTG E51-2009 [25], “Test Method of Inorganic Binder Stabilizing Material for Highway Engineering”, the optimum water content and maximum dry density for each proportion outlined in Table 2 and Table 3 were determined through a compaction test. The inner diameter of the compaction cylinder is 100 mm, the height of the cylinder is 127 mm, the number of hammering layers is 5, and each layer is hammered 27 times. The results of the compaction test of each ratio are presented in Table 4.

### 2.4. Preparation Process of HPG-CSSB Sample

**Figure 2:**
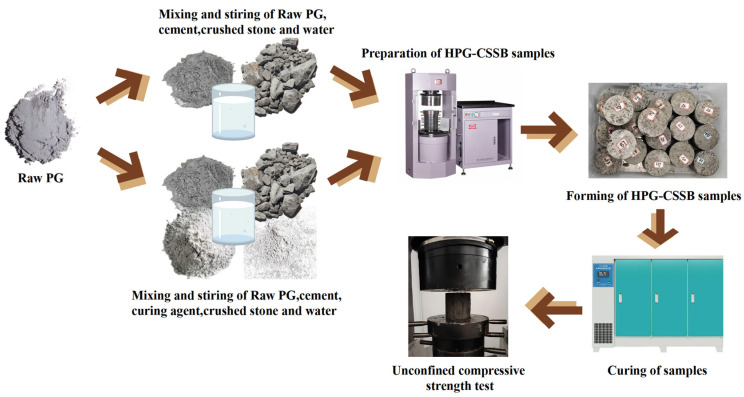
Preparation process of HPG-CSSB samples.

In accordance with T 0843-2009, “Sample Preparation Methods of Materials Stabilized with Inorganic Binders”, and T 0845-2009, “Curing Test Methods of Materials Stabilized with Inorganic Binders”, as outlined in JTG E51-2009 [25], a cylindrical test mold with diameter × height = ϕ100 mm × 100 mm was constructed, and graded crushed stone that had been moistened for one hour was along with the inorganic binders, which were then mixed thoroughly and added to the mold. The samples were prepared using a pressure testing machine and placed in a plastic bag after demolding. The samples were then placed in a curing box at 20 ± 2 °C and 95% humidity for curing. On the day prior to the testing date, the samples were removed from the plastic bag and immersed in water for 24 h. Thereafter, the relevant tests and characterizations were carried out. Figure 2 illustrates the preparation process.

### 2.5. Analysis and Characterization Methods

In accordance with JTG E51-2009 [25], “Test Method of Inorganic Binder Stabilizing Material for Highway Engineering”, a microcomputer-controlled oil-electric hybrid servo pressure testing machine (Wance HCT306E, Shenzhen, China) was used for the unconfined compressive strength testing. Nine samples were molded for strength determination for each mixture, and the water content in the phosphogypsum (PG) was determined using the drying method. Water stability was assessed by calculating the ratio of the UCS of water-saturated cured samples to the UCS of conventionally cured samples. Prior to testing, the surface of the block sample is ground to a flat surface. The powdered sample is then ground to the requisite fineness for each test. Thereafter, individual samples are prepared for microscopic testing. Particle size analysis of PG was conducted using laser diffraction methods (LD) (Malvern Mastersizer 3000+, Worcestershire, UK). Scanning electron microscopy (SEM) and energy-dispersive X-ray spectroscopy (EDS) (Zeiss Sigma-300, Oberkochen, Germany) were employed for observing the microstructure. Phase analysis was performed using X-ray diffraction (XRD) (Rigaku Ultima IV, Tokyo, Japan) over a scanning range of 5–75°, and the Jade 6.0 software package was used for quantitative and qualitative analysis of the XRD data. Chemical structure analysis was carried out using Fourier-transform infrared spectroscopy (FTIR) (Thermo Fisher IS5, Waltham, MA, USA). Leaching toxicity testing was conducted in accordance with HJ 557-2010 [26], “Solid Waste—Extraction Procedure for Leaching Toxicity—Horizontal Vibration Method”; and the concentration of leaching elements was measured using inductively coupled plasma mass spectrometry (ICP-MS) (Agilent 7800 ICP-MS, Santa Clara, CA, USA).

## 3. Discussion of Results

### 3.1. Raw Materials Analysis

**Figure 3:**
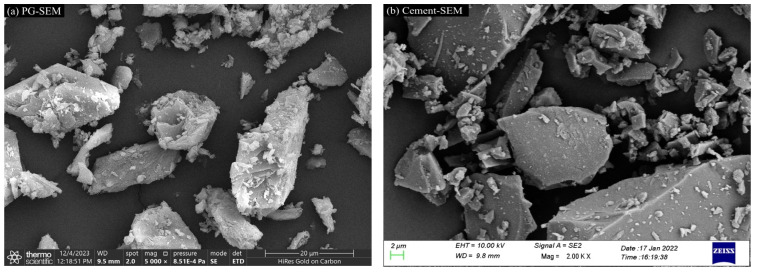
SEM of raw materials.

**Figure 4:**
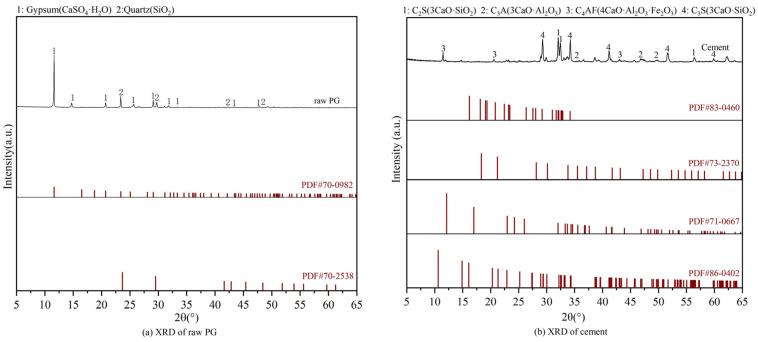
XRD of raw materials.

**Figure 5:**
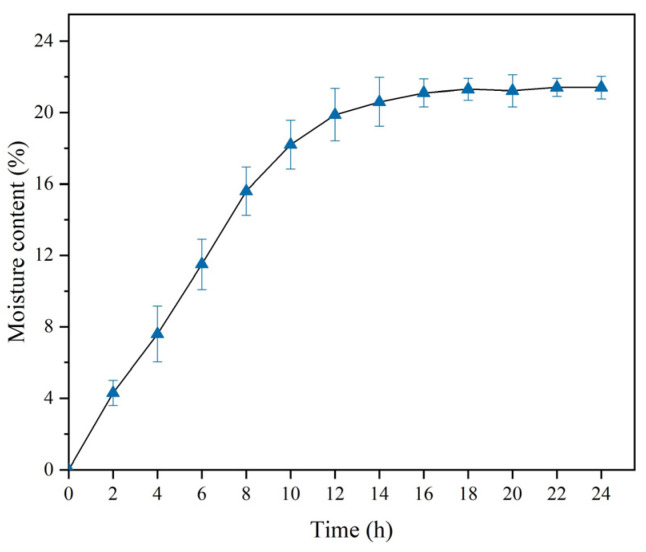
Water content of PG versus time.

**Figure 6:**
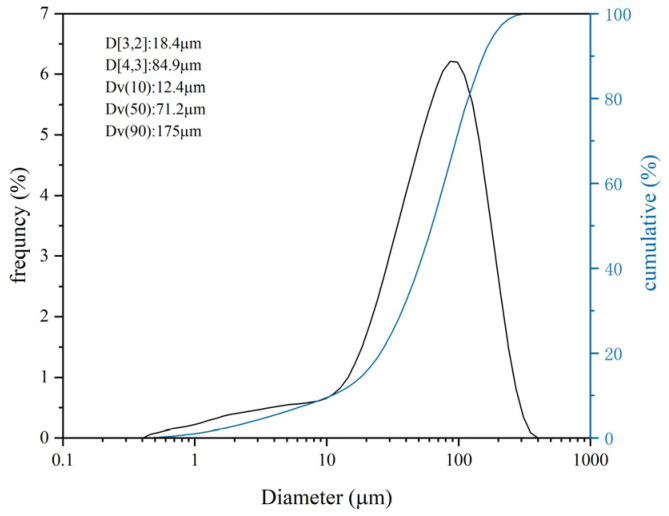
Particle-size distribution of PG.

The scanning electron microscope (SEM) and X-ray diffraction (XRD) scans of PG and cement are presented in Figure 3 and Figure 4, respectively. The chemical compositions are provided in Table 5. The PG was found to consist primarily of dihydrate gypsum (CaSO_4_·2H_2_O) and quartz (SiO_2_), with narrow, sharp characteristic peaks that indicate high crystallinity and large grains; the cement was primarily composed of C_2_S(3CaO·SiO_2_), C_3_A(3CaO·Al_2_O_3_), C_4_AF, and C_3_S. The water content in the PG was determined by the drying method, as the crystalline water in the PG is easily removed at high temperatures. The raw PG was subjected to drying in an oven maintained at a constant temperature of 70 °C for 24 h, with the mass of the sample weighed every two hours, as illustrated in Figure 5. After 16 h, the moisture content exhibited a tendency to stabilize, and the final moisture content of the PG was determined to be 21%. The particle size distribution of the PG is illustrated in Figure 6. The distribution range is approximately 0.3 μm to 400 μm with a median particle size of 71.2 μm.

### 3.2. Unconfined Compressive Strength Analysis

**Figure 7:**
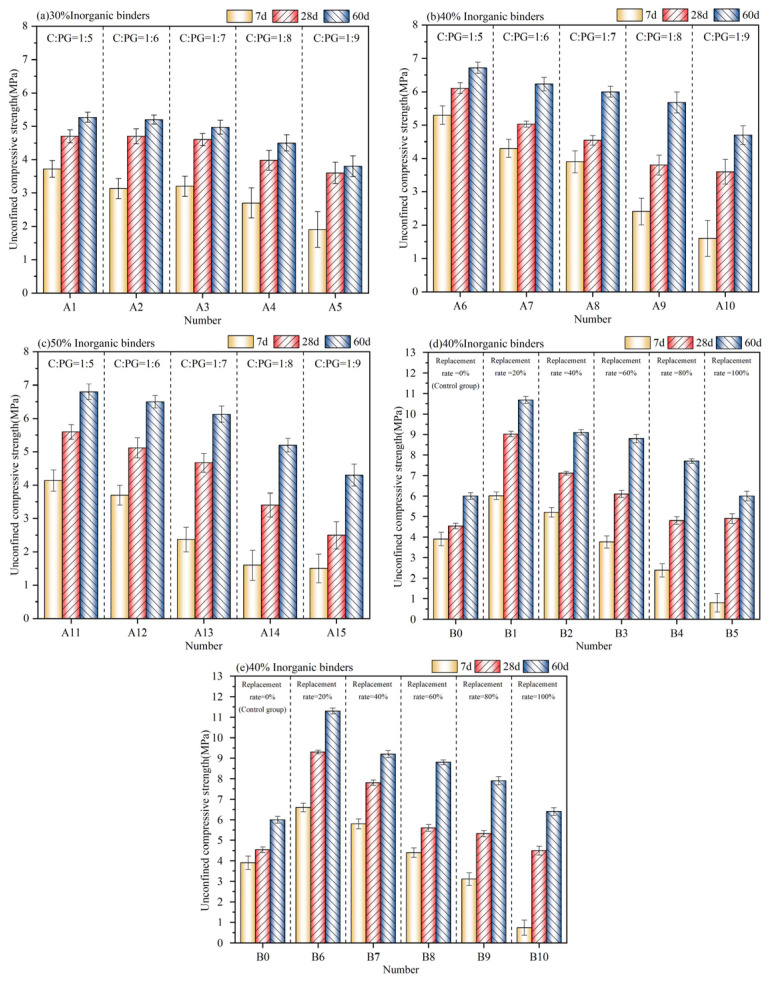
Unconfined compressive strength of HPG-CSSB at 7 d, 28 d, and 60 d.

Figure 7a–c illustrates the 7 d, 28 d, and 60 d UCS of HPG-CSSB at varying cement, PG, and crushed stone admixtures. Overall, the UCS of the samples demonstrated a tendency to increase and then decrease as the percentage of inorganic binders increased. It was observed that as the cement PG ratio increased, the UCS of the samples also increased. Among samples with the same cement PG ratio, the 7 d UCS with 40% inorganic binders is higher than those with 30% and 50% inorganic binders. This is due to the fact that at the early stage in the context of maintenance, it is observed that a greater proportion of PG is not converted into ettringite (AFt) but rather utilized as filler aggregate within the mixture. The structure of the mixture can be classified as belonging to the suspension and compacting types. At this juncture, the structure of the mixture is in a suspended state, with an inorganic binder proportion of 40%, which is conducive to the formation of a stable skeleton structure. PG lacks cementing properties; thus, it primarily reacts with 3CaO·Al_2_O_3_ (C_3_A) in cement clinker to form AFt, thereby enhancing strength. Consequently, increasing the cement dosage while maintaining the same percentage of inorganic binders results in a higher 7 d UCS for the sample. The high dosage of PG will impede the cement hydration process, resulting in an incomplete cement hydration reaction in the initial stages of the system. This, in turn, affects the 7 d UCS to a certain extent. However, as the curing period progresses, the C_3_A continues to react with PG, resulting in the generation of a substantial quantity of AFt. Additionally, the hydrated calcium silicate (C-S-H) released from 2CaO·SiO_2_ (C_2_S) in the cement clinker at the later stage of hydration contributes to an enhancement in the specimen strength. This is evidenced by an observed increase in the 28 d and 60 d UCS. Furthermore, the increase in UCS of the samples with a lower cement PG ratio is more pronounced in the later stage, which results in a relatively minor discrepancy in 60 d UCS of different cement PG ratios. Given that the cement dosage is typically not in excess of 5% in the context of road subgrade engineering, the aggregate accounted for 60%, the inorganic binders for 40%, and the cement PG ratio of 1:7 for A8 represents the optimum mix ratio. Under this mix ratio, the UCS with different curing ages was 3.9 MPa, 4.9 MPa, and 6.0 MPa at 7 d, 28 d, and 60 d, respectively. In the current Chinese specification, cement stabilized materials are primarily assessed based on their 7 d UCS, with the UCS of A8 capable of attaining the requisite strength standard for highway base layer under both medium and light traffic loading levels.

Figure 7d,e depict the 7 d, 28 d, and 60 d UCS of HPG-CSSB with inorganic binders at 40% and PG dosage at 35%, using CA1 and CA2 to replace the cement with 0%, 20%, 40%, 60%, 80%, and 100% replacement, respectively. The control group designated B0, consisted of samples with the same inorganic binders ratio but without a curing agent, which was identical to that used for A8. As illustrated in Figure 6, the 7 d, 28 d, and 60 d UCS of the samples with a 20% replacement rate exhibited a notable enhancement in comparison to the samples with no curing agent added. The 7 d, 28 d, and 60 d UCS of B1 demonstrated an enhancement of 53.8% (6.0 MPa), 100.0% (9.0 MPa), and 78.3% (10.7 MPa), respectively, in comparison to B0. Additionally, the 7 d, 28 d, and 60 d UCS of B6 were increased by 69.2% (6.6 MPa), 104.8% (9.3 MPa), and 88.3% (11.3 MPa), respectively, in comparison to B0. Both B1 and B6 are capable of attaining the requisite strength standard for the highway base layer under extremely heavy traffic loading levels. Furthermore, the effect of CA2 on the UCS at different curing ages was more pronounced than that of CA1. It is noteworthy that following the substitution rate exceeding 20%, the UCS of samples at all ages exhibited a gradual decline with the increase in substitution rate, particularly evident in the 7 d UCS. This suggests that the primary mechanism of the curing agent within the system is to facilitate the hydration reaction of cement, thereby producing a greater quantity of hydration products within the specimen and enhancing its strength. For CA1 and CA2 to demonstrate an optimum curing effect, it is essential that they are situated within an environment that contains an adequate quantity of cement.

### 3.3. Water Stability Analysis

**Figure 8:**
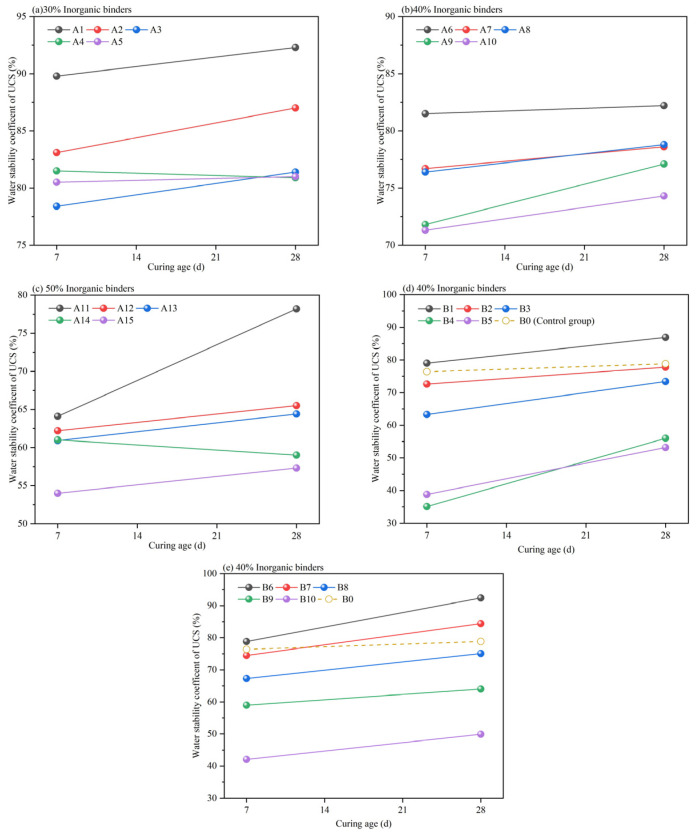
Water stability coefficient of HPG-CSSB at 7 d and 28 d.

The water stability index is a key indicator for assessing the durability of subgrade materials. Figure 8 illustrates the water stability coefficients of the 7 d and 28 d UCS of HPG-CSSB. In this test, the water stability coefficients were determined by calculating the ratio of the UCS of the water-saturated curing samples to that of the conventional curing samples. As PG is a highly absorbent powder with no cementitious properties, higher dosing will result in poorer water stability of the samples after immersion in water. The water stability of HPG-CSSB is primarily attributable to the hydration products of cement. The C-S-H gel and AFt generated by cement hydration serve to densify the pores between the aggregates, thereby reducing the degree of water intrusion into the samples. Consequently, the water stability coefficient of the samples with a higher cement dosage in the same percentage of inorganic binders is also higher. The water stability coefficient of each mix ratio increases with age due to the slow initial hydration rate of the stabilized material, leading to enhanced structural stability and reduced external water influence.

As illustrated in Figure 8d,e, the incorporation of two curing agents at a ratio of 20% cement replacement has been observed to enhance the water stability coefficients of the original fitment B0. This enhancement is particularly pronounced at the 28 d, with the 28 d water stability coefficient of B1 mixed with CA1 exhibiting an 86.9% increase and the 28 d water stability coefficient of B6 mixed with CA2 demonstrating a 92.4% surge. These observations indicate a notable improvement in water resistance performance. This is due to the fact that the addition of the curing agent results in the generation of a greater quantity of hydration gel with AFt, which serves to enhance the internal pore structure while simultaneously increasing the compressive strength [27]. The generation of substantial quantities of needle and rod AFt leads to the formation of a skeletal structure within the interstitial spaces between cement particles, hydration products, gypsum, and aggregates. The process results in a notable enhancement in the density of the pore structure and a considerable improvement in water resistance, as evidenced by SEM-EDS of Section 3.6.

### 3.4. XRD Analysis

The XRD patterns and quantitative XRD analysis of HPG-CSSB 28d at varying coordination ratios are illustrated in Figure 8a,b, respectively. Figure 8 illustrates the primary hydration products of HPG-CSSB, which include Ettringite (Ca_6_Al_2_(SO_4_)_3_(OH)_12_·26H_2_O), C-S-H gel, Quartz (SiO_2_), and Yeelimite (Ca_4_Al_6_O_12_(SO_4_)), and Yeelimite and Ettringite belong to the crystal structure of Ettringite [28]. Given that the C-(A)-S-H hydration product is amorphous in nature, it is not discernible in XRD analysis [29]. Quartz and gypsum (CaSO_4_·2H_2_O) are unreacted phases of raw PG. The presence of Ca(OH)_2_ was not detected, which was attributed to the conversion of Ca(OH)_2_ to C-S-H by volcanic ash reaction with SiO_2_ during the later stages of hydration.

**Figure 9:**
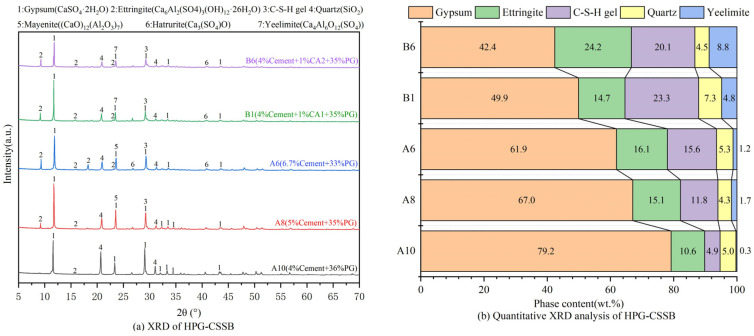
XRD and quantitative analysis of HPG-CSSB with 40% inorganic binders at 28 d.

As illustrated in Figure 9, the Gypsum diffraction peaks at 11.72°, 22.5°, and 33.5° (2θ) exhibited greater intensity for A10 with 4.0% cement doping in the absence of a curing agent. Conversely, the Ettringite diffraction peaks observed at 8.94°, 15.62°, and 22.68° (2θ) and the C-S-H diffraction peak at 29.32° (2θ) displayed diminished intensity. As the quantity of cement doping increased, the diffraction peaks of Ettringite and C-S-H continued to strengthen, while those of gypsum continued to weaken. This demonstrates that the doping of cement affects the intensity and content of the diffraction peaks of Ettringite and C-S-H. Furthermore, the increase in cement doping contributes to the generation of hydration products in the HPG-CSSB system, which is consistent with the results presented in Section 3.2 of the UCS results. The diffraction peaks of the hydration products of B1 and B6 with an added curing agent were significantly enhanced in comparison to B0, and the peak of Gypsum was significantly weakened. This indicates that the slag in the curing agent, along with other alkaline substances, stimulated the activity of the cement, which was beneficial to the sustained hydration of the cement. This resulted in the production of more C-S-H gel in the system, along with more PG being converted to Ettringite. The quantitative XRD results for Ettringite and C-S-H indicate that the hydration effect of B6 was superior to that of B1. This is primarily attributable to the higher Metakaolin content of CA2 compared to CA1, which resulted in a more robust volcanic ash activity within the reaction environment. In contrast, Ettringite represents the primary crystalline hydration product within the curing system [30]. The curing agent, however, induces a greater transformation of gypsum within the system. Consequently, the diffraction peak intensity of Ettringite in B6 is markedly higher than that of B1, while the diffraction peak of gypsum is comparatively lower.

### 3.5. FTIR Analysis

**Figure 10:**
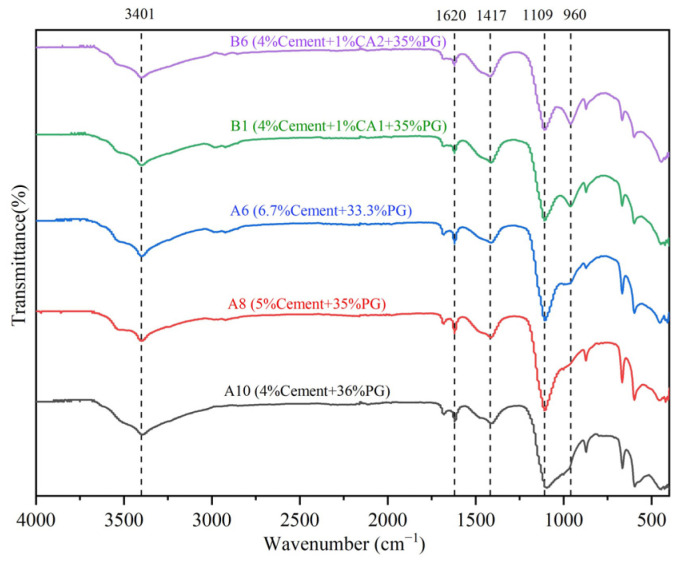
FTIR spectra of HPG-CSSB with 40% inorganic binders at 28 d.

Figure 10 illustrates the Fourier transform infrared spectra (FTIR) of HPG-CSSB with varying mix ratios. The FTIR of the samples without added curing agent exhibited comparable absorption peaks, suggesting that the distinct cement PG ratios did not exert a substantial influence on the nature of the hydration products, which predominantly comprised C-S-H gels and AFt crystals. In the FTIR of B1 and B6 with the addition of the curing agent, an asymmetric stretching vibrational band of Si-O-Al at 960 cm^−1^ can be observed [31], which indicates the generation of C-(A)-S-H gel [32]. This is due to the high volcanic ash activity provided by the curing agent to the system, which caused Ca^2+^ in PG to react with Al_2_O_3_ and SiO_2_ to generate C-(A)-S-H gels. The bending vibration peaks of OH^−^ at 3401 cm^−1^ and 1620 cm^−1^ are primarily attributed to crystal water or bound water in PG [33]. The observed weakening of the hydroxyl absorption peaks suggests an increased involvement of PG in the hydration reaction. The asymmetric telescopic vibration band of CO_3_^2−^ at 1417 cm^−1^ indicates that the sample has undergone a carbonation reaction with CO_2_ in the air. The lower peak heights of the absorption peaks of the Si-O asymmetric stretching vibrational bands at 1083 cm^−1^–1122 cm^−1^ indicate that after the introduction of the curing agent, additional hydration products, AFt crystals, and C-S-H gels were produced in the system, which would weaken the bond energy and lead to the breakage of the Si-O bonds [34,35].

### 3.6. SEM-EDS Analysis

**Figure 11:**
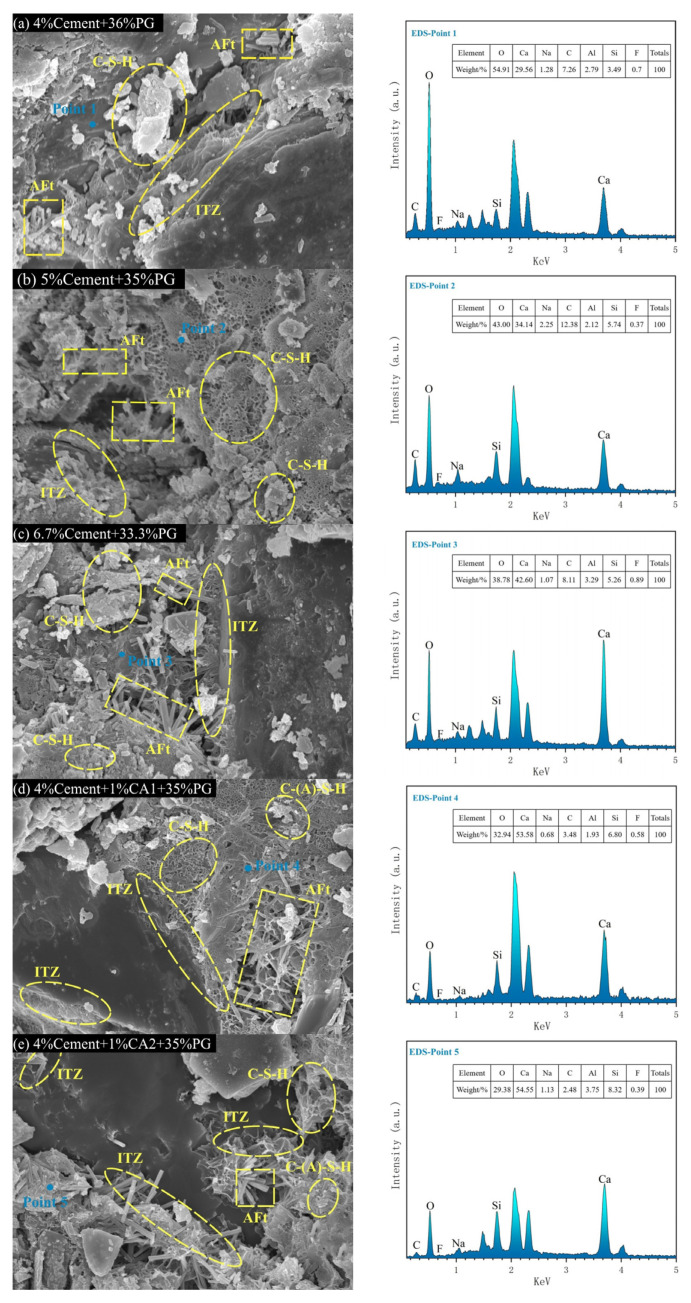
SEM-EDS of HPG-CSSB with 40% inorganic binders at 28 d.

Figure 11a–c depicts the SEM-EDS images of the interfacial transition zone (ITZ) of A10, A8, and A6 at 28 d. It can be observed that the production of hydration products occurs within the internal interfacial zone of PG cement stabilized materials. These products primarily consist of cluster-like and foil-like C-S-H gels, plate-like rhombic or short columnar gypsum crystals, and needle-and-rod AFt crystals. As illustrated in Figure 11a, the generation of hydration products is minimal, and the density of the connection between the hydration products and the interface zone is low. Additionally, there is considerable pore space, which can be attributed to the high dosage of PG, which provides a weakly acidic environment. This environment slows the hydration of cement particles in the early stages of the reaction process. Furthermore, in sulfate-rich environments, cement particles rapidly generate dense AFt on the surface, impeding the subsequent hydration of cement particles. After 28 days of curing, the ITZ contains only a small amount of clustered C-S-H gel, which is insufficient to fill the pores between aggregate and gypsum crystals, resulting in a loose structure and poor strength properties. The elevation of cement dosing resulted in a notable increase in the number of foil-like C-S-H gels and needle-like and rod-like AFt observed in Figure 11b. These AFt were found to be interspersed within the C-S-H gels, forming a skeleton. However, the pore gaps remained present within the structure. The ITZ in Figure 11c is partially wrapped by the C-S-H gel and tightly bonded to the interlaced AFt skeleton, which effectively fills the inter-aggregate pore gaps. As illustrated in the EDS diagram, the primary constituents of the hydrogel are calcium, silicon, oxygen, aluminum, and other elements. In combination with XRD and SEM, this indicates that the hydration products are primarily C-S-H and AFt. The high oxygen content and low calcium and silicon content in EDS point 1 suggest that the quantity of cement hydration products in the material is minimal, and there is also a considerable amount of uninvolved in the reaction, namely CaSO_4_·2H_2_O. The EDS points 2 and 3 display an increase in the elemental content of Ca, Si, and Al, indicating that the C-S-H and AFt content is enhanced with the elevation of cement dosing. This phenomenon is also observed in the SEM.

The SEM-EDS images of the ITZ of B1 and B6 28 d are presented in Figure 11d,e, while Figure 11b serves as a control group in the absence of a hardener. In comparison to Figure 11b, a considerable number of needle-and-rod AFt, in addition to foil-like C-S-H gel that is firmly attached to or encases the ITZ, and flocculent hydrated calcium silica-aluminate (C-(A)-S-H) gels that are partially formed by the hydration of the curing agent, can be observed in Figures. The alkaline slag-base polymer curing agent partially neutralized the weakly acidic environment provided by the highly dosed PG. This process not only stimulates the hydration activity of the cement but also causes a significant reaction of the large amount of Ca^2+^ supplied by the PG with the SiO_2_ and Al_2_O_3_ in the slag, producing C-S-H gels and C-(A)-S-H gels through a second hydration distinct from the hydration of the cement. This subsequently binds with the ITZ, wrapping around the AFt skeleton in a more stable manner, and through adhesion, immobilizes it, which results in a reduction in internal pores and an overall increase in density. This is reflected in the macroscopic performance, with the UCS of B1 and B6 exhibiting a notable improvement compared to that of B0. In comparison to Figure 11d, an increased amount of AFt is visible in Figure 11e. This is attributed to the fact that the metakaolin in CA2 stimulates the generation of AFt in the hydration products, thereby further enhancing the mechanical properties of the specimens through the reinforcement of the AFt skeleton. This is not a result that can be obtained with CA1. The elevated Ca and Si levels in EDS points 4 and 5 relative to EDS point 2 indicate that the incorporation of the curing agent results in enhanced C-S-H and C-(A)-S-H formation, which is corroborated by the observations presented in Section 3.4 and Section 3.5.

The absence of a P element in the five EDS points illustrated in Figure 11a–e indicates that phosphorus-containing impurities in PG can be adsorbed by C-S-H and immobilized within the gel lattice. The presence of a minor quantity of F element in the EDS data implies that the encapsulation and ionic conversion of hydrated gel can effectively immobilize fluoride in PG, with only a limited portion of the fluorine-containing pollutants escaping onto the gel surface. This finding is in accordance with the leaching toxicity concentration data presented in Table 6.

### 3.7. Leaching Toxicity Analysis

Given that the leaching toxicity of raw PG markedly exceeded the threshold set by the current Chinese effluent discharge standard, a more comprehensive assessment of the leaching toxicity of HPG-CSSB and an in-depth investigation into the solidification mechanism of pollutants by the HPG-CSSB system are imperative to facilitate the resource utilization of PG. Table 6 illustrates the leaching concentrations of F^−^, PO_4_^3−^, and heavy metals at 28 d for raw PG and HPG-CSSB with varying mix ratios. Table 6 illustrates that the F^−^ leaching concentration of raw PG was 144.143 mg/L, while the F^−^ leaching concentration of HPG-CSSB ranged from 5.434 to 11.311 mg/L, representing a reduction of approximately 93.18%. The PO_4_^3−^ leaching concentration of raw PG concentration was 315.923 mg/L, while the PO_4_^3−^ leaching concentration of HPG-CSSB ranged from 0.085 to 0.963 mg/L, representing a reduction of approximately 99.94%. Of the samples, A8, A6, B1, and B6 met the leaching toxicity concentration requirements for F^−^ and PO_4_^3−^. In the context of wastewater discharge standards, the heavy metals present in raw PG were predominantly As^3+^, with a leaching concentration of 1.628 mg/L, which exceeded the 0.5 mg/L standard value specified in GB 8978-1996 Class A. In comparison, the heavy metal leaching concentration of HPG-CSSB was 0.5 mg/L, which was in alignment with the aforementioned standard. The leaching concentration of As^3+^ in HPG-CSSB was reduced by approximately 99.49%, and the leaching concentration of the remaining heavy metals was also diminished to a notable extent. These results align with the relevant standards set forth in China.

During the production of raw PG, the high concentration of phosphoric acid solution makes Ca^2+^ difficult to diffuse, resulting in the distribution of impurity particles on the surface of PG crystals. These soluble impurities can be immobilized or removed by ion substitution and physical adsorption in HPG-CSSB. During the hydration process of the HPG-CSSB system, a substantial quantity of Ca^2+^ is released by gypsum and cement in an alkaline environment. This Ca^2+^ can convert soluble F^−^ and PO_4_^3−^ into insoluble calcium fluoride and orthophosphate. Additionally, heavy metals react with OH^−^ to produce hydroxide precipitation [38]. The C-S-H gel produced by the system will form on the surface of PG crystals and will adsorb and immobilize toxic and hazardous substances in soluble impurities, demonstrating a high degree of targeting and efficacy. The adsorption capacity of these substances in PG will be enhanced with the increase in C-S-H gel. The reaction equation is as follows (1)–(6):

CaSO_4_·2H_2_O + 2F^−^ → CaF_2_ + SO_4_^2−^ + 2H_2_O (1)

(Ca^2+^, Ni^2+^, Mg^2+^) + 2OH^−^ → (Ca, Ni, Mg)(OH)_2_ (2)

(OH)_2_As^3+^ + 3OH^−^ → As(OH)_3_ (3)

(Ca^2+^, Ni^2+^, Mg^2+^) + 2F^−^ → (Ca, Ni, Mg)F_2_ (4)

(Ca, Ni^2+^, Mg)(OH)_2_ + PO_4_^3−^ → (Ca, Ni, Mg)PO_3_(OH)·2H_2_O (5)

(Ca^2+^, Ni^2+^, Mg^2+^) + PO_4_^3−^ → (Ca, Ni, Mg)_3_(PO_4_)_2_ (6)

## 4. Conclusions

A novel pavement base layer, designated HPG-CSSB, was formulated through the incorporation of a high dosage of PG, cement, and a self-developed curing agent with graded crushed stone. Subsequent to an exhaustive analysis of the mechanical properties, hydration characteristics, microstructure, and leaching toxicity of the HPG-CSSB samples, the following conclusions were reached:

(1) The optimum mix ratio in HPG-CSSB was determined to be 4% cement, 1% CA2, 35% cement, and 60% graded crushed stone. The UCS of the samples at 7 d, 28 d, and 60 d under this mix ratio were 6.6 MPa, 9.3 MPa, and 11.3 MPa, respectively. Notably, the 7 d UCS met the criterion for cement-stabilized base layers of highways and primary roads under very heavy traffic, as outlined in JTG/T F20-2015 [39]. The water stability coefficients of the specimens at 7 d and 28 d were 78.8% and 92.4%, respectively. The material demonstrated robust strength properties and excellent water resistance.
(2) The slag-based geopolymer curing agent has been demonstrated to stimulate the hydration activity of HPG-CSSB. In the absence of a curing agent, the high dosage of gypsum impeded cement hydration, resulting in a considerable quantity of PG remaining untransformed into AFt. Following the introduction of a modest quantity of curing agent, the concentration and stability of C-S-H gel and AFt crystals within the material were markedly enhanced, and the optimal hydration of HPG-CSSB based on optimal mixing ratios was observed from XRD, FTIR, and SEM-EDS.
(3) The hydration mechanism of HPG-CSSB: The hydration reaction results in the rapid generation of AFt in cement and PG, where C_3_A is present and SO_4_^2−^ is present in PG. C_3_S in cement continues to undergo hydration, resulting in the generation of C-S-H gel and Ca(OH)_2_. The generated Ca(OH)_2_ is then converted to C-S-H through a volcanic ash reaction with PG and SiO_2_ in cement. The curing agent provides high volcanic ash activity, facilitating the hydration of Ca^2+^ in the system and SiO_2_ and Al_2_O_3_ in the curing agent twice to generate C-S-H and C-(A)-S-H. Subsequently, C_2_S in the cement continues to hydrate to generate C-S-H.
(4) The hydration products in the HPG-CSSB system demonstrated a high capacity for target immobilization of toxic and hazardous substances present in PG. The system facilitated ion exchange and chemical precipitation of pollutants through the hydration-generated C-S-H gel and AFt, converting soluble F^−^, PO_4_^3−^, and Ni^2+^ to insoluble substances. The leaching toxicity of the HPG-CSSB was found to comply with the current Chinese wastewater discharge standards.

## Figures and Tables

**Table 1:** Gradation design of aggregates.

Sieve Aperture Size/mm	The Pass Rate of Each Sieve Aperture in the Gradation Design/%
19	100
16	90.5
13.2	81
9.5	65.5
4.75	40
2.36	24.8
1.18	14.5
0.6	8
0.3	1.5

**Table 2:** Mix design devoid of a curing agent.

Number	Graded Crushed Stone Dosage	Cement Dosage	PG Dosage	Cement PG Ratio
A1	70%	5.0%	25.0%	1:5
A2	70%	4.3%	25.7%	1:6
A3	70%	3.8%	26.3%	1:7
A4	70%	3.3%	26.7%	1:8
A5	70%	3.0%	27.0%	1:9
A6	60%	6.7%	33.3%	1:5
A7	60%	5.7%	34.3%	1:6
A8	60%	5.0%	35.0%	1:7
A9	60%	4.4%	35.6%	1:8
A10	60%	4.0%	36.0%	1:9
A11	50%	8.3%	41.7%	1:5
A12	50%	7.1%	42.9%	1:6
A13	50%	6.3%	43.7%	1:7
A14	50%	5.6%	44.4%	1:8
A15	50%	5.0%	45.0%	1:9

**Table 3:** Mix design with curing agent.

Number	Graded Crushed Stone Dosage	Curing Agent Type	Cement Dosage	Curing Agent Dosage	PG Dosage	
B0 (Control group)	60%	-	5.0%	-	35.0%	
B1	60%	CA1	4.0%	1.0%	35.0%	
B2	60%		3.0%	2.0%	35.0%	
B3	60%		2.0%	3.0%	35.0%	
B4	60%		1.0%	4.0%	35.0%	
B5	60%		0%	5.0%	35.0%	
B6	60%	CA2	4.0%	1.0%	35.0%	
B7	60%		3.0%	2.0%	35.0%	
B8	60%		2.0%	3.0%	35.0%	
B9	60%		1.0%	4.0%	35.0%	
B10	60%		0%	5.0%	35.0%	

**Table 4:** Proctor compaction test results.

Number	Optimum Water Content (%)	Maximum Dry Density (g/cm^3^)
A1	9.41	2.285
A2	9.67	2.251
A3	9.83	2.237
A4	10.03	2.219
A5	10.2	2.203
A6	11.34	2.089
A7	11.6	2.078
A8	11.77	2.071
A9	11.89	2.056
A10	12.02	2.053
A11	13.21	1.860
A12	13.45	1.841
A13	13.68	1.828
A14	13.8	1.823
A15	13.95	1.812
B1	11.71	2.077
B2	11.67	2.080
B3	11.59	2.086
B4	11.55	2.091
B5	11.48	2.099
B6	11.74	2.073
B7	11.68	2.079
B8	11.63	2.083
B9	11.58	2.086
B10	11.51	2.095

**Table 5:** Chemical compositions of cement and PG (wt.%).

Chemical Compositions	SO_3_	CaO	SiO_2_	F	P_2_O_5_	Al_2_O_3_	Fe_2_O_3_	Na_2_O	MgO	Others
PG	52.64	38.36	4.15	1.73	1.51	0.57	0.21	0.21	0.19	0.43
Cement	1.25	54.43	22.71	-	-	9.86	4.75	0.86	2.28	3.86

**Table 6:** HPG-CSSB pollutants leaching and integrated wastewater discharge standard.

Sample	Leaching Concentration (mg/L)							
	F^−^	PO_4_^3−^	Pb^2+^	Cr^6+^	Total Cr	As^3+^	Ba^2+^	Ni^2+^
Raw PG	144.143	315.923	0.019	-	0.046	1.628	0.069	0.054
A10	11.311	0.963	0.021	-	0.042	0.018	0.042	0.012
A8	8.878	0.257	0.018	-	0.037	0.012	0.031	0.008
A6	7.159	0.128	0.007	-	0.033	-	0.022	-
B1	7.120	0.196	0.012	-	0.041	0.003	0.033	0.013
B6	5.434	0.085	-	-	0.023	-	0.019	0.008
GB 8978-1996 [36] Class A	10	0.5	1.0	0.5	1.5	0.5	-	1.0
GB 5085.3-2007 [37]	100	-	5	5	15	5	100	5

## Data Availability

The original contributions presented in the study are included in the article, further inquiries can be directed to the corresponding author.
